# Statin Treatment and Mortality in Community-Dwelling Frail Older Patients with Diabetes Mellitus: A Retrospective Observational Study

**DOI:** 10.1371/journal.pone.0130946

**Published:** 2015-06-25

**Authors:** Alberto Pilotto, Francesco Panza, Massimiliano Copetti, Matteo Simonato, Daniele Sancarlo, Pietro Gallina, Timo Strandberg, MPI_AGE Project Investigators

**Affiliations:** 1 Department of OrthoGeriatrics, Rehabilitation and Stabilization, Frailty Area, E.O. Galliera Hospital, National Relevance and High Specialization Hospital, Genova, Italy; 2 Geriatrics Unit, Azienda ULSS 16 Padova, S. Antonio Hospital, Padova, Italy; 3 Neurodegenerative Disease Unit, Department of Basic Medicine, Neuroscience, and Sense Organs, University of Bari Aldo Moro, Bari, Italy; 4 Gerontology-Geriatrics Research Laboratory, IRCCS Casa Sollievo della Sofferenza, San Giovanni Rotondo, Italy; 5 Unit of Biostatistics, IRCCS Casa Sollievo della Sofferenza, San Giovanni Rotondo, Foggia, Italy; 6 Health Directorate, Azienda ULSS 16, Padova, Italy; 7 Geriatric Clinic, Department of Medicine, University of Helsinki, Helsinki, Finland; 8 Institute of Health Sciences/Geriatrics, University of Oulu, and Oulu University Hospital, Oulu, Finland; University of Verona, Ospedale Civile Maggiore, ITALY

## Abstract

**Background:**

Older adults are often excluded from clinical trials. Decision making for administration of statins to older patients with diabetes mellitus (DM) is under debate, particularly in frail older patients with comorbidity and high mortality risk. We tested the hypothesis that statin treatment in older patients with DM was differentially effective across strata of mortality risk assessed by the Multidimensional Prognostic Index (MPI), based on information collected with the Standardized Multidimensional Assessment Schedule for Adults and Aged Persons (SVaMA).

**Methods:**

In this retrospective observational study, we estimated the mortality risk in 1712 community-dwelling subjects with DM ≥ 65 years who underwent a SVaMA evaluation to establish accessibility to homecare services/nursing home admission from 2005 to 2013 in the Padova Health District, Italy. Mild (MPI-SVaMA-1), moderate (MPI-SVaMA-2), and high (MPI-SVaMA-3) risk of mortality at baseline and propensity score-adjusted hazard ratios (HR) of three-year mortality were calculated according to statin treatment.

**Results:**

Higher MPI-SVaMA scores were associated with lower rates of statin treatment (MPI-SVaMA-1 = 39% vs MPI-SVaMA-2 = 36% vs MPI-SVaMA-3 = 24.9%. p<0.001) and higher three-year mortality (MPI-SVaMA-1 = 12.9% vs MPI-SVaMA-2 = 24% vs MPI-SVaMA-3 = 34.4%, p<0.001). After adjustment for propensity score quintiles, statin treatment was significantly associated with lower three-year mortality irrespective of MPI-SVaMA group (interaction test p = 0.303). HRs [95% confidence interval (CI)] were 0.19 (0.14–0.27), 0.28 (0.21–0.36), and 0.26 (0.20–0.34) in the MPI-SVaMA-1, MPI-SVaMA-2, and MPI-SVaMA-3 groups, respectively. Subgroup analyses showed that statin treatment was also beneficial irrespective of age. HRs (95% CI) were 0.21 (0.15–0.31), 0.26 (0.20–0.33), and 0.26 (0.20–0.35) among patients aged 65–74, 75–84, and ≥ 85 years, respectively (interaction test p=0.812).

**Conclusions:**

Statin treatment was significantly associated with reduced three-year mortality independently of age and multidimensional impairment in community-dwelling frail older patients with DM.

## Introduction

Clinical trial data of treatment with 3-hydroxy-3-methylglutaryl-CoA reductase inhibitors (statins) in persons over 75–80 years are scarce [1, 2], and the benefits and potential harms of statin treatment are frequently disputed. This especially applies to persons without cardiovascular disease [3] and to persons who are frail and multimorbid and therefore assessed to be at heightened risk of statin adverse effects [4].

The recently discovered increased risk of diabetes mellitus (DM) during statin treatment, suggested by observational studies [5], randomized clinical trials (RCTs) [6], and large meta-analyses [7], may have further fuelled these concerns. In particular, in a large meta-analysis on 13 statin trials with 91140 participants, meta-regression showed that risk of development of DM with statins was highest in trials with older participants [7], a group for which the absolute benefit of statin treatment would also be greater. Actually, there are very limited data assessing the impact of statins on older patients with DM [8] and the available evidence on other outcomes such as frailty, physical and cognitive function, and institutionalization is mixed [9–12]. On the other hand, there is also a danger that older patients with DM are unnecessarily deprived of potentially useful therapy [13, 14]. Recent studies have shown that clinical decision making on statin prescription in older patients only seldom considers risk stratification [15, 16], resulting in many hospitalized or community-dwelling older patients with DM not receiving statin therapy. Certainly, the clinical decision about statin therapy in older patients with DM is very challenging and should take into account both the risk of complications and the expected future survival time [1].

Many lines of research demonstrated that mortality risk stratification in older patients should be based on information on comorbidity and functional status [17], and it is best performed using a multidimensional Comprehensive Geriatric Assessment (CGA) that integrate information of several domains of health and function [18]. Recently, a Multidimensional Prognostic Index (MPI) derived from a standardized CGA has been developed and validated for mortality risk assessment in several independent cohorts of hospitalized [19] and community-dwelling older subjects [20] with acute or chronic diseases. The aim of the present study was to test the hypothesis that statin treatment in community-dwelling frail older patients with DM is differentially effective across strata of mortality risk.

## Methods

### Study Population

This was a retrospective observational study conducted according to the World Medical Association's 2008 Declaration of Helsinki, the guidelines for Good Clinical Practice, and the Strengthening the Reporting of Observational Studies in Epidemiology (STROBE) guidelines [21]. All consecutive community-dwelling older subjects aged 65 years and older who underwent a CGA-based multidimensional assessment according to the Standardized Multidimensional Assessment Schedule for Adults and Aged Persons (Scheda per la Valutazione Multidimensionale delle persone adulte e Anziane) (SVaMA) [20] from January 1^st^ 2005 to December 31^st^ 2013 were screened for inclusion in the study. Inclusion criteria were: [1] diagnosis of DM according to the ICD9 250 and subgroups or according to the main diagnosis record T90 (“diabetes mellitus”) of the SVAMA; [2] a SVaMA evaluation within 2 months from the date of the first registration of the DM diagnosis in the database. The Institutional Review Board of the Social and Health-Care Local Unit (Unità Locale Socio Sanitaria, ULSS) 16, Padova, Italy approved this retrospective observational study. Informed consent was given by participants who underwent SVaMA evaluation and/or their proxies for their clinical records to be used in clinical studies. All patient records and information were anonymized and de-identified prior to the analysis. For statin users, the “enrollment” was defined as the first statin prescription which succeed the date of the registration of the DM diagnosis. For statin non-users, the “enrollment” was defined as the date of the SVaMA completion which succeed the date of the first registered DM diagnosis in the database. If the date of SVaMA completion preceded the date of the DM diagnosis registration, the time interval between these dates was lower than 2 months. Subjects were followed for a mean follow-up of 3.20±2.76 years. Vital status was assessed by consulting the Registry Offices of the cities in which the patients were residents at the time of the evaluation. Dates of death were identified from death certificates. All the data regarding the evaluations were extracted from the Administrative Repository Database of the ULSS 16, Padova, Italy.

### The Multidimensional Prognostic Index (MPI) Based on the SVaMA

The SVaMA is the officially recommended multidimensional assessment schedule used since 2000 by the health personnel of the National Health Care System (NHS) to perform a multidimensional assessment in community-dwelling older persons introduced by the Veneto Regional Health System since 2000 to establish accessibility to some health care resources (homecare services or nursing home admission) [20]. In order to calculate the MPI, the following domains of the SVaMA were considered: 1) age, 2) sex, 3) main diagnosis, 4) Nursing Care Needs (VIP) evaluated according to a validated numeric scale including 11 items that estimated the nursing care needs of the older subject; 5) Cognitive status (VCOG), evaluated by the Short Portable Mental Status Questionnaire (SPMSQ); 6) the pressure sores risk (VPIA), evaluated by the Exton-Smith Scale; 7) the activities of daily living (VADL) and 8) mobility (VMOB) evaluated by the Barthel Index; 9) social support (VSOC), evaluated by a numeric scale of 16 items that explores the presence of a support network during the day and the night. The SVaMA instrument (Italian version) is available on-line at the following address: http://www.uneba.org/regione-veneto-nuova-svama-e-nuova-svamdi/.

To calculate the MPI from the SVaMA, a weighted sum of each individual domain (**D**_i_) was computed (raw formula). Weights (**S**_i_) were estimated from a multivariate Cox proportional hazard model for 1-year mortality prediction. Each weighted sum (**R** = ∑(**S**_i_^**.**^ **D**_i_)) was then normalized into a range that varies from 0 (lowest risk) to 1 (highest risk), subtracting the observed raw minimum value and then dividing such difference by the observed range (minimum to maximum span). The MPI-SVaMA was expressed as a continuous value from 0 (lower risk) to 1.0 (higher risk of mortality). The RECursive Partition and AMalgamation (RECPAM) algorithm [22] was used to identify subgroups of patients at different risks for mortality [20]. The following cut offs were estimated for the normalized MPI-SVaMA one-year mortality prediction: 0–0.33 (MPI-SVaMA-1 mild risk), 0.34–0.47 (MPI-SVaMA-2 moderate risk), 0.48–1.0 (MPI-SVaMA-3 severe risk). To calculate the MPI-SVaMA at 1 month and at 1 year, software for Windows may be downloaded (available for free) at the following address: http://www.mpiage.eu/home/about-mpi-svama (English version). Further information on reliability, accuracy, calibration and validation of the MPI based on the SVaMA can be found elsewhere [20].

### Drug Treatment Assessment

Our cohort was linked to the Pharmaceutical Prescription database of the Azienda ULSS 16 Padova to extract the individual medication use. Statin and other drug prescriptions were determined according to the Anatomical Therapeutic Chemical (ATC) codes. Statin prescription was determined by C10 ATC code. Individuals were considered statin-users if they received statin prescriptions after the first registered evidence of the DM diagnosis. In the present study, we included all statin-users who achieved a treatment adherence coverage, i.e., the ratio between treatment duration (in days) and individual follow-up duration (in days) of at least 100% for the first year, 90% and 80% when considering the outcome at two and three years of follow-up, respectively. We defined statin non-users, the older individuals who never received statin prescriptions. As a proxy of patients’ polypharmacy, we used the mean monthly past treatment rate defined as the total number of drug boxes taken before the enrollment divided by the total number of months between the first prescription and the enrollment.

### Statistical Analysis

Baseline characteristics were reported as frequencies (percentages) and mean±standard deviation (SD), for categorical and continuous variables, respectively. Comparisons between men and women were performed using Pearson chi-square test and Mann-Whitney U test, whereas tests for linear trend across MPI grades were performed using ANOVA models or Mantel-Haenszel chi-square tests for continuous and categorical variables, respectively. Mortality incidence rates were computed as the number of new events per 100 person-years and compared using a Poisson regression models. To control possible confounding effects on the association between statin treatment and mortality risk, the propensity score (PS) methodology was applied [23]. PS logistic regression models were built to predict the probability to receive statin according to all variables used for the calculation of MPI-SVaMA at treatment assignment: age, sex, VIP, VCOG, VPIA, VADL, VMOB, VSOC, the main diagnoses of fractures, cancer, dementia, stroke, hypokinetic syndrome and cardiovascular, respiratory neurological or other diseases and the past treatment rate (in tertiles). PS logistic models were selected in a step-wise fashion, and model-building stopped when adequate balance of covariates was achieved [23]. Residual imbalances of covariates in PS quintiles were assessed at each step with a two-way analysis of variance (ANOVA) where each confounder was considered as an outcome and PS quintiles and treatment as factors. Overlapping of PS between treatment and control groups was also checked, and non-overlapping subjects were excluded from the analyses. Separate PS logistic models were run for the overall sample and MPI grade subgroups. Multivariable and PS-quintiles adjusted Cox regression models were used to assess the effect of statin use on three-years mortality, and results were reported as hazard ratios (HRs) along with their 95% confidence intervals (95% CIs). In addition, to check the robustness of our findings, a 5 to 1 greedy 1:1 PS matching algorithm was performed. PS 1:1 matching identified a unique matched control for each treated patient according to their PS. Adequacy of covariate balance in the matched sample was eventually assessed with McNemar or Wilcoxon’s signed rank test. For the overall sample and for specific MPI-SVaMA grade subgroups, adjusted HRs of statin use for three-year mortality were reported along with total number of events, total subjects per group and mortality rates. Multivariable models included: statin treatment, age, sex, the main diagnoses, all domains of MPI-SVaMA and the past treatment rate as covariates. As the PS matched sample did not consist of independent observations, a marginal survival model with robust standard errors was used. P-values assessing the presence of a heterogeneous effect of statin treatment between MPI-SVaMA risk subgroups were also calculated and reported. Two-sided *P*-values <0.05 were considered statistically significant. All the analyses were performed using SAS Release 9.1.3 (SAS Institute, Cary, NC).

## Results

### Characteristics of the Study Population

Initially, 1899 subjects aged 65 years and older with a diagnosis of DM were screened. Of these, 187 subjects (9.8%) were excluded because their SVaMA evaluation was not performed within 2 months from the date of the first registration of the DM diagnosis (86 subjects) or due to a statin low treatment adherence coverage during follow-up as defined above (101 subjects). Therefore, the study population included 1712 patients, 740 men (43.2%) and 972 women (56.8%) with a mean age of 81.1±7.33 years. Men were younger (79.01±7.1 vs 82.71±7.3 years, p<0.001), had higher MPI mean values (0.46±0.2 vs 0.35±0.16, p<0.001), higher VIP (10.32±9.3 vs 8.31±8.5, p<0.001), higher prevalence of cancer (27.98% vs 11.32%, p<0.001), and showed significantly higher mortality incidence rates at three years of follow-up (25.8% vs 18.8%, p<0.001) than women. Women were significantly more cognitively impaired as measured by VCOG (men = 4.39±3.6 vs women = 5.16±3.5, p<0.001) and had higher prevalence of dementia (men = 16.08% vs women = 23.77%, p<0.001). No significant differences between sexes were observed in the overall past treatment rates. The proportion of patients starting statin treatment was higher in men than in women (65.94% vs 59.2%, p<0.001).

Table 1 shows the characteristics of patients divided according to their MPI-SVaMA grade: 603 patients (35.2%) were in MPI-SVaMA-1 mild-risk, 662 patients (38.7%) in MPI-SVaMA-2 moderate-risk and 447 patients (26.1%) in MPI-SVaMA-3 severe-risk of mortality. Patients with higher MPI-SVaMA values were more likely to be males (p for trend <0.001) and older (p for trend <0.001) and had significantly higher VADL, VCOG, VIP, VMOB, VPIA, and VSOC scores (all domains p for trend <0.001). Three-year mortality incidence rates were MPI-SVaMA-1: 12.9%, MPI-SVaMA-2: 24.0%, and MPI-SVaMA-3: 34.4% (p for trend <0.001).

**Table 1:** Baseline characteristics of community-dwelling older patients with diabetes mellitus divided according to their Multidimensional Prognostic Index (MPI) grade based on the Standardized Multidimensional Assessment Schedule for Adults and Aged Persons (SVaMA).

	All (n = 1712)	MPI-SVaMA-1 Mild risk (n = 603)	MPI-SVaMA-2 Moderate risk (n = 662)	MPI-SVaMA-3 Severe risk (n = 447)	p-value (test for trend)
Patients (%)	100%	35.2%	38.7%	26.1%	——
Age at SVaMA evalutation (years)	81.11±7.33	79.13±6.84	81.99±7.40	82.47±7.30	<0.001
Sex (n males, %)	740 (43.22)	188 (31.18)	275 (41.54)	277 (61.97)	<0.001
VADL	40.23±19.12	21.25±15.09	46.77±12.82	56.15±6.82	<0.001
VCOG	4.83±3.56	3.28±3.12	4.97±3.43	6.71±3.36	<0.001
VIP	9.18±8.91	4.04±4.95	7.64±6.74	18.40±8.94	<0.001
VMOB	29.08±12.50	16.54±11.09	33.92±7.50	38.84±2.62	<0.001
VPIA	4.80±6.35	0.09±0.97	4.38±5.46	11.76±5.51	<0.001
VSOC	156.45±69.05	138.62±69.96	164.18±65.79	169.06±67.76	<0.001
Number of medications*	31.87±45.69	32.51±45.98	32.30±45.83	30.39±45.15	0.282
**Main diagnosis**					
Fractures (n,%)	22 (1.29%)	5 (0.83%)	12 (1.81%)	5 (1.12%)	<0.001^#^
Cancer (n,%)	317 (18.52%)	95 (15.75%)	116 (17.52%)	106 (23.71%)	
Dementia (n,%)	350 (20.44%)	144 (23.88%)	141 (21.30%)	65 (14.54%)	
Stroke (n,%)	138 (8.06%)	30 (4.98%)	57 (8.61%)	51 (11.41%)	
Cardiovascular disease (n,%)	209 (12.21%)	78 (12.94%)	91 (13.75%)	40 (8.95%)	
Respiratory disease (n,%)	42 (2.45%)	16 (2.65%)	14 (2.11%)	12 (2.68%)	
Neurologic disease (n,%)	61 (3.56%)	33 (5.47%)	16 (2.42%)	12 (2.68%)	
Ipokinetic syndrome (n,%)	188 (10.98%)	37 (6.14%)	86 (12.99%)	65 (14.54%)	
Other diseases (n,%)	385 (22.49%)	165 (27.36%)	129 (19.49%)	91 (20.36%)	
Follow-up time (years)	3.20±2.76	4.06±2.88	2.93±2.65	2.45±2.45	<0.001
Mortality at 1 year (ev/py, ir %)^	442/1377 (32.1%)	83/536 (15.5%)	186/523 (35.6%)	173/319 (54.3%)	<0.001
Mortality at 2 years (ev/py, ir %)^	599/2445 (24.5%)	136/986 (13.8%)	240/916 (26.2%)	223/543 (41.0%)	<0.001
Mortality at 3 years (ev/py, ir %)^	719/3317 (21.7%)	176/1368 (12.9%)	295/1228 (24.0%)	248/721 (34.4%)	<0.001

VADL: activities of daily living; VCOG: cognitive status; VIP: Nursing Care Needs; VMOB: mobility; VPIA: pressure sores risk; VSOC: social support

* Number of all medications per month, taken before the patient’s enrollment

^ ev/py: events/person-years, ir%: incidence rate (number of events per 100 person-years)

Overall, 1064 patients with DM (62.15% of the total study population with DM) were treated with statins. The treated patients (Table 2) were younger (p = 0.001), had lower impairment in VCOG (p = 0.002), VPIA (p = 0.02), VADL (p = 0.001), VMOB (p<0.001) scores and lower MPI-SVaMA values (p<0.001) than untreated patients. Moreover, patients treated with statins were more frequently in the MPI-SVaMA-1 group (39% vs 29%, p<0.001) and in the highest tertile of medication number (3-tertile, 40.23% vs. 22.53%, p<0.001) than patients untreated with statins.

**Table 2:** Pre-matching baseline characteristics of community-dwelling older patients with diabetes mellitus according to statin use.

	Not treated (n = 648)	Treated (n = 1064)	p-value	Standardized mean difference
Patients (%)	37.85%	62.15%	——	——
Age at SVaMA evaluation (years)	83.42±7.43	79.70±6.90	<0.001	-51.779
Sex (n males, %)	252 (38.89%)	488 (45.86%)	0.005	14.152
VCOG	5.16±3.51	4.62±3.58	0.002	-15.250
VIP	8.98±8.55	9.31±9.13	0.790	3.776
VPIA	5.23±6.53	4.53±6.22	0.022	-10.929
VADL	42.29±18.35	38.97±19.47	0.001	-17.589
VMOB	30.59±11.88	28.16±12.77	<0.001	-19.691
VSOC	163.79±67.90	151.98±69.39	0.001	-17.205
Fractures (n,%)	13 (2.01)	9 (0.85)	0.039	-9.7983
Cancer (n,%)	57 (8.80)	260 (24.44)	<0.001	42.976
Dementia (n,%)	125 (19.29)	225 (21.15)	0.356	4.6236
Stroke (n,%)	52 (8.02)	86 (8.08)	0.966	0.2132
Cardiovascular disease (n,%)	73 (11.27)	136 (12.78)	0.353	4.6641
Respiratory disease (n,%)	13 (2.01)	29 (2.73)	0.351	4.7347
Neurologic disease (n,%)	14 (2.16)	47 (4.42)	0.015	12.6795
Ipokinetic syndrome (n,%)	92 (14.20)	96 (9.02)	<0.001	-16.2073
Other diseases (n,%)	209 (32.25)	176 (16.54)	<0.001	-37.2113
MPI-SVaMA (continuous)	0.40±0.11	0.38±0.12	<0.001	-16.481
MPI-SVaMA -1 mild risk (n,%)	188 (29.01%)	415 (39.00%)	<0.001	21.209
MPI- SVaMA-2 moderate risk (n,%)	278 (42.90%)	384 (36.09%)		-13.967
MPI- SVaMA-3 severe risk (n,%)	182 (28.09%)	265 (24.91%)		-7.211
Number of medications* (1°tertile-Low; n,%)	298 (45.99%)	275 (25.85%)	<0.001	-42.940
Number of medications* (2°tertile-Med; n,%)	204 (31.48%)	361 (33.93%)		5.218
Number of medications* (3°tertile-High; n,%)	146 (22.53%)	428 (40.23%)		38.845

VCOG: cognitive status; VIP: Nursing Care Needs; VPIA: pressure sores risk; VADL: activities of daily living; VMOB: mobility; VSOC: social support; MPI: Multidimensional Prognostic Index

MPI-SVaMA: Multidimensional Prognostic Index based on the Standardized Multidimensional Assessment Schedule for Adults and Aged Persons

* Number of all medications prescribed within one year before patient’s enrollment

### Association of Statin Treatment with Mortality

In the whole study population, multivariable analysis (adjusted for age, sex, main diagnoses, all MPI-SVaMA domains, and past treatment) showed that, during the three years of follow-up, statin treatment was associated with lower mortality risk (Table 3). Statin treatment was significantly associated with lower three-year mortality risk, irrespective of the MPI-SVaMA grade (Table 3). In the whole population, the significant association between statin treatment and lower three-year mortality was confirmed even after the adjustment for PS quintiles. Similarly, statin treatment was significantly associated with lower mortality risk within each class of MPI-SVaMA. The HRs (95%CI) were: 0.19 (0.14–0.27), 0.28 (0.21–0.36), and 0.26 (0.20–0.34) in patients in MPI-SVaMA-1, MPI-SVaMA-2, and MPI-SVaMA-3 respectively, although no differential effectiveness on mortality risk was found across MPI-SVaMA groups (interaction test p = 0.303). Finally, the association between statin treatment and reduction of mortality was also independent of age. The PS-quintiles adjusted HRs (95% CI) were: 0.21 (0.15–0.31), 0.26 (0.20–0.33), and 0.26 (0.20–0.35) in patients aged from 65 to 74 years, from 75 to 84 years, and ≥ 85 years, respectively (interaction test p = 0.812). The PS-based greedy matching algorithm successfully matched 547 of 1064 treated patients. Adequacy of covariate balance in the matched sample was shown in S1 Table. Results of statin treatment effects from marginal univariate Cox regression models, with robust standard errors, were fully overlapping with those reported in Table 3 (S2 Table).

**Table 3:** Overall and subgroup analyses for community-dwelling older patients with diabetes mellitus statin users vs. non-users: multivariate and propensity score (PS) quintiles adjusted models.

						Three-year Mortality rate (n° events per 100 person-years)				Multivariate models*			PS quintiles adjusted models		
						All	Statin use		Change^	HR	95% CI	p-value	HR	95% CI	p-value
		Events		Patients	Person-years		No	Yes							
**MPI-SVaMA GRADE**	MPI-SVaMA-1	176		603	1368	12.9	29.1	8.1	-21.0	0.13	0.09–0.19	<0.001	0.19	0.14–0.27	<0.001
	mild risk														
	MPI-SVaMA-2	295		662	1228	24.0	48.2	14.4	-33.8	0.24	0.18–0.31	<0.001	0.28	0.21–0.36	<0.001
	moderate risk														
	MPI-SVaMA-3	248		447	721	34.4	88.0	19.2	-68.8	0.23	0.17–0.31	<0.001	0.26	0.20–0.34	<0.001
	severe risk														
**AGE**	65–74.9 years	140		376	796	17.6	44.9	12.4	-32.5	0.11	0.07–0.17	<0.001	0.21	0.15–0.31	<0.001
	75–84.9 years	320		800	1628	19.7	45.1	12.8	-32.3	0.21	0.16–0.27	<0.001	0.26	0.20–0.33	<0.001
	≥85 years	259		536	893	29.0	53.8	13.5	-40.3	0.24	0.18–0.32	<0.001	0.26	0.20–0.35	<0.001
	All	719		1712	3317	21.7	48.7	12.8	-35.9	0.21	0.18–0.25	<0.001	0.25	0.21–0.30	<0.001

MPI-SVaMA: Multidimensional Prognostic Index based on the Standardized Multidimensional Assessment Schedule for Adults and Aged Persons

* Models were adjusted for: age at SVaMA evaluation, sex, Nursing Care Needs (VIP), cognitive status (VCOG), pressure sores risk (VPIA), activities of daily living (VADL), mobility (VMOB), social support (VSOC) (all MPI-SVaMA domains), the needing of care assistants, the main diagnoses of fractures, cancer, dementia, stroke, hypokinetic syndrome and cardiovascular, respiratory neurological or other diseases and number of all medications prescribed within one year before patient’s enrollment (tertiles);

^Difference of mortality rates between statin users vs. non-users

## Discussion

The present retrospective observational study demonstrated that in the overall study population of community-dwelling older patients with DM, statin treatment was associated with a reduced three-year mortality. Importantly, the present findings demonstrated that a severely compromised health and functional status (reflected by the MPI-SVaMA), or a very old age, did not affect the association between statin treatment with reduced mortality. Therefore, even a severe multidimensional impairment or a very advanced age should not be considered as contraindications to statin treatment in older patients with DM.

Indeed, while many studies have established statin efficacy in cardiovascular prevention in middle-aged people, only few studies have suggested a reduced mortality in older patients treated with statins [1]. Moreover, no previous study has explored the interaction of statin treatment with individual mortality risk in frail older patients with DM. In agreement with recent studies [8], we found that statin treatment was underrepresented in this older population with DM, i.e., the prevalence of statin use was only 22.7%. The patients in the present study belonged to a population of frail older patients who underwent a CGA based on the SVaMA in order to assess needs for support (homecare services or nursing home admission) by the NHS. Therefore, we cannot exclude that the low prevalence of statin use may reflect the reluctance of physicians to treat older patients with frequent clinical, functional, and social impairments, that is frailty. Actually, notwithstanding indications, the statin-treated patients were significantly younger, had lower functional, cognitive, and clinical impairments, and they had a significant lower mortality risk than untreated patients. To address this selection bias, PS methods were used to define cohorts which only differed for the treatment with statins. Both the PS-adjusted models and the analyses within the PS-matched cohorts confirmed that the benefit from statin treatment was evident in DM patients independently of the MPI risk grade. The subgroup analyses for heterogeneity, moreover, showed that the effect of statin treatment was not significantly different among patients with different mortality risks. Thus, patients who were less frequently prescribed statins, with higher MPI-SVaMA scores, showed a larger clinical benefit in terms of reduced mortality. Consequently, statin treatment reduced their risk to the same level of older patients at lower mortality risk, as assessed with the MPI score. Although we did not have laboratory assessment to support a cholesterol-related role of statins on mortality, a growing body of evidence suggested that total cholesterol level has little relationship to mortality in older patients with statin treatment [24]. This may indicate that anti-inflammatory, antioxidant, or other “pleiotropic” effects may play a role for reduction of mortality [4]. Conversely, there was a well-established correlation linking low total cholesterol, low-density lipoprotein (LDL) cholesterol, and high-density lipoprotein (HDL) cholesterol to increased mortality in older age [25]. Furthermore, multidimensional impairment assessed by the MPI was also associated with low total, LDL, and HDL cholesterol [26], suggesting that higher total cholesterol may be paradoxically associated with better survival as an example of “reverse epidemiology” [4]. At present, the impact of statin treatment in older subjects on outcomes such as frailty, physical and cognitive function, and institutionalization is controversial [9–12]. In particular, in a prospective study of more than 25,000 women 65 years old or older who were initially free of frailty, current use of statin medications was not signiﬁcantly related to the development of frailty at three-year follow-up [10]. Furthermore, in a recent population-base study, frail men were more likely to be institutionalized and die than non-frail men, independent of their statin exposure [12].

To evaluate the mortality risk in this older population, we adopted the MPI [19] based on the SVaMA [20], a well calibrated and highly accurate predictor of mortality in this age group [17, 27]. The MPI-SVaMA domain variables included multidimensional and integrated information on clinical, functional, cognitive, and social status of patients. This approach identified frailty as a composition of multisystemic changes occurring in older subjects that may determine an increased risk for adverse health outcomes, including death [18]. Accordingly, the MPI score was very effective in predicting mortality in different settings. Indeed, a large multicentre study performed in over 2000 hospitalized older patients demonstrated that MPI had a significantly higher predictive power for all-cause mortality compared with three other widely diffused frailty instruments [28]. Furthermore, we obtained consistent results with various analytical approaches, including careful PS adjustment and matching. We must also acknowledge limitations of the study. Firstly, we considered the effectiveness of statins only in terms of reduced all-cause mortality, not taking into account causes of deaths nor nonfatal events. Nevertheless, considering the net clinical benefit, a reduced total mortality is probably very relevant in older subjects with a reduced life expectancy. Secondly, the present findings were observational and noninterventional. Given the lack of randomization to statin treatment, we cannot exclude that a better chance of survival was considered among the motivation to start statins. However, no RCTs of statins have so far included persons older than 82 years at baseline [1] and we therefore need observational studies in frail older subjects to cumulate evidence for treatment decisions. This is supported by a very recent Cochrane review assessing the impact of study design on the effect measures estimated that demonstrated no significant differences between observational studies and RCTs, regardless of specific observational study design, heterogeneity, or inclusion of studies of pharmacological interventions [29]. Furthermore, we did not have laboratory variables (serum lipids, cholesterol, triglycerides, glucose, or glycosylated haemoglobin) available for our analysis. the number of medications is the same among the three groups of risk (table). In the present study, among the three MPI- SVaMA grades linked to a different mortality risk, no differences were found for the number of medications. In the severe risk group (MPI- SVaMA-3), one would expect a higher number of medications suggesting probably an undertreatment in this group of subjects probably due to the reluctance of physicians to expose to polypharmacy the more impaired older frail patients. Finally, since the follow-up of these patients was limited to three years, we cannot exclude that significant differences in effectiveness among patients with different mortality risk could emerge with longer follow-up. Nevertheless, three years can be assumed to be a substantial follow-up period in this older and frail population with high mortality risk.

The present findings suggested that statin treatment could be implemented also in older frail patients with DM, with some consequences for the multidimensional management of patients requiring support from the NHS. The reduced mortality associated with statin use in these community-dwelling frail older patients with DM may suggest a significant impact of statin treatment also in patients requiring homecare services or nursing home admission. This could have important policy implications for the burden that frail older patients with DM may pose on the NHS and healthcare systems worldwide. Only 31.8% of patients who had a cardiac hospitalization and then were discharged to a nursing home received a statin [30]. However, further real-world trials specifically designed for frail older patients with DM are needed to confirm the impact of statins on survival and other clinical outcomes in this particular subgroup.

### Ethical Approval

The study was approved by the Institutional Review Board of the Social and Health-Care Local Unit (Unità Locale Socio Sanitaria, ULSS) 16, Padova, Italy.

## Supporting Information

S1 Table Post-matching baseline characteristics of community-dwelling older patients with diabetes mellitus according to statin use. SVaMA: Standardized Multidimensional Assessment Schedule for Adults and Aged Persons; VCOG: cognitive status; VIP: Nursing Care Needs; VPIA: pressure sores risk; VADL: activities of daily living; VMOB: mobility; VSOC: social support; MPI: Multidimensional Prognostic Index. * Number of all medications prescribed within one year before patient’s enrollment.
(DOC)

S2 Table Overall and subgroup analyses for community-dwelling older patients with diabetes mellitus statin users vs. non-users (propensity score 1:1 matching models). MPI-SVaMA: Multidimensional Prognostic Index- Standardized Multidimensional Assessment Schedule for Adults and Aged Persons Patients were matched, using a 5 to 1 greedy 1:1 PS matching algorithm for: age at SVaMA evaluation, sex, Nursing Care Needs (VIP), cognitive status (VCOG), pressure sores risk (VPIA), activities of daily living (VADL), mobility (VMOB), social support (VSOC) (all MPI-SVaMA domains), the needing of care assistants, the main diagnoses of fractures, cancer, dementia, stroke, hypokinetic syndrome and cardiovascular, respiratory neurological or other diseases and number of all medications prescribed within one year before patient’s enrollment (tertiles). *Testing whether the effect of statins on mortality risk was differential within MPI-SVaMA grades (effect modifier).
(DOC)
