# Supplementary material for: Statin Treatment and Mortality in Community-Dwelling Frail Older Patients with Diabetes Mellitus: A Retrospective Observational Study
Source: PLoS One. 2015 Jun 25;10(6):e0130946. doi: 10.1371/journal.pone.0130946 (PMC4482517; doi:10.1371/journal.pone.0130946)
Supplement: S1 Table — SVaMA: Standardized Multidimensional Assessment Schedule for Adults and Aged Persons; VCOG: cognitive status; VIP: Nursing Care Needs; VPIA: pressure sores risk; VADL: activities of daily living; VMOB: mobility; VSOC: social support; MPI: Multidimensional Prognostic Index. * Number of all medications prescribed within one year before patient’s enrollment. (DOC) [file pone.0130946.s001.doc]

**S1 Table. This is the S1 Table title.** Post-matching baseline characteristics of community-dwelling older patients with diabetes mellitus according to statin use.

|  | **Not treated (n=547)** | **Treated**  **(n=547)** | **p-value** | **Standardized mean difference** |
| --- | --- | --- | --- | --- |
|  |  |  |  |  |
| Patients (%) | 50.0% | 50.0% | ---- | ---- |
| Age at SVaMA evaluation (years) | 82.29±7.20 | 82.47±6.14 | 0.798 | 2.60667 |
| Sex (n males, %) | 227 (41.50) | 224 (40.95) | 0.854 | -1.1142 |
| VCOG | 5.10±3.58 | 5.13±3.55 | 0.819 | 0.87223 |
| VIP | 9.20±8.78 | 9.16±9.08 | 0.672 | -0.42977 |
| VPIA | 5.13±6.53 | 5.05±6.47 | 0.916 | -1.20956 |
| VADL | 41.52±18.85 | 42.00±18.21 | 0.680 | 2.5743 |
| VMOB | 29.95±12.19 | 30.30±11.43 | 0.605 | 2.92399 |
| VSOC | 160.72±68.21 | 160.96±69.68 | 0.883 | 0.34204 |
| Fractures (n,%) | 7 (1.28) | 9 (1.65) | 0.617 | 3.04608 |
| Cancer (n,%) | 57 (10.42) | 69 (12.61) | 0.163 | 6.87614 |
| Dementia (n,%) | 114 (20.84) | 112 (20.48) | 0.879 | -0.90313 |
| Stroke (n,%) | 50 (9.14) | 45 (8.23) | 0.596 | -3.24648 |
| Cardiovascular disease (n,%) | 72 (13.16) | 74 (13.53) | 0.862 | 1.07519 |
| Respiratory disease (n,%) | 13 (2.38) | 10 (1.83) | 0.532 | -3.8236 |
| Neurologic disease (n,%) | 14 (2.56) | 11 (2.01) | 0.549 | -3.67084 |
| Ipokinetic syndrome (n,%) | 73 (13.35) | 78 (14.26) | 0.649 | 2.65029 |
| Other diseases (n,%) | 147 (26.87) | 139 (25.41) | 0.547 | -3.32883 |
| MPI-SVaMA (continuous) | 0.39±0.12 | 0.39±0.12 | 0.849 | 0.99559 |
| MPI-SVaMA-1 mild risk (n,%) | 167 (30.53) | 186 (34.00) | 0.486 | 7.4351 |
| MPI-SVaMA-2 moderate risk (n,%) | 229 (41.86) | 205 (37.48) |  | -8.97763 |
| MPI-SVaMA-3 severe risk (n,%) | 151 (27.61) | 156 (28.52) |  | 2.03454 |
| Number of medications* (1°tertile-Low; n, %) | 218 (39.85) | 211 (38.57) | 0.236 | -2.62136 |
| Number of medications* (2°tertile-Med; n, %) | 185 (33.82) | 202 (36.93) |  | 6.50344 |
| Number of medications* (3°tertile-High; n, %) | 144 (26.33) | 134 (24.50) |  | -4.20009 |

This is the S1 Table legend.

SVaMA: Standardized Multidimensional Assessment Schedule for Adults and Aged Persons; VCOG: cognitive status; VIP: Nursing Care Needs; VPIA: pressure sores risk; VADL: activities of daily living; VMOB: mobility; VSOC: social support; MPI: Multidimensional Prognostic Index

* Number of all medications prescribed within one year before patient’s enrollment
