# Supplementary material for: Statin Treatment and Mortality in Community-Dwelling Frail Older Patients with Diabetes Mellitus: A Retrospective Observational Study
Source: PLoS One. 2015 Jun 25;10(6):e0130946. doi: 10.1371/journal.pone.0130946 (PMC4482517; doi:10.1371/journal.pone.0130946)
Supplement: S2 Table — MPI-SVaMA: Multidimensional Prognostic Index- Standardized Multidimensional Assessment Schedule for Adults and Aged Persons Patients were matched, using a 5 to 1 greedy 1:1 PS matching algorithm for: age at SVaMA evaluation, sex, Nursing Care Needs (VIP), cognitive status (VCOG), pressure sores risk (VPIA), activities of daily living (VADL), mobility (VMOB), social support (VSOC) (all MPI-SVaMA domains), the needing of care assistants, the main diagnoses of fractures, cancer, dementia, stroke, hypokinetic syndrome and cardiovascular, respiratory neurological or other diseases and number of all medications prescribed within one year before patient’s enrollment (tertiles). *Testing whether the effect of statins on mortality risk was differential within MPI-SVaMA grades (effect modifier). (DOC) [file pone.0130946.s002.doc]

**S2 Table. This is the S2 Table title.** Overall and subgroup analyses for community-dwelling older patients with diabetes mellitus statin users vs. non-users (propensity score 1:1 matching models).

|  |  | **Events** | **Patients** | **HR** | **95% CI** | **p-value** | **Statins and MPI-SVaMA interaction test (p-value)*** |
| --- | --- | --- | --- | --- | --- | --- | --- |
|  |  |  |  |  |  |  |  |
| **1 year** | MPI-SVaMA-1  mild risk | 60 | 353 | 0.21 | 0.11-0.37 | <0.001 | 0.740 |
|  | MPI-SVaMA-2 moderate risk | 144 | 434 | 0.24 | 0.17-0.36 | <0.001 |  |
|  | MPI-SVaMA-3  severe risk | 127 | 307 | 0.21 | 0.14-0.31 | <0.001 |  |
|  | All | 331 | 1094 | 0.23 | 0.18-0.29 | <0.001 |  |
| **2 years** | MPI-SVaMA-1  mild risk | 88 | 353 | 0.21 | 0.13-0.34 | <0.001 | 0.733 |
|  | MPI-SVaMA-2 moderate risk | 176 | 434 | 0.27 | 0.19-0.38 | <0.001 |  |
|  | MPI-SVaMA-3  severe risk | 163 | 307 | 0.26 | 0.19-0.36 | <0.001 |  |
|  | All | 427 | 1094 | 0.26 | 0.21-0.32 | <0.001 |  |
| **3 years** | MPI-SVaMA-1  mild risk | 106 | 353 | 0.20 | 0.13-0.31 | <0.001 | 0.489 |
|  | MPI-SVaMA-2 moderate risk | 200 | 434 | 0.28 | 0.21-0.38 | <0.001 |  |
|  | MPI-SVaMA-3  severe risk | 180 | 307 | 0.27 | 0.20-0.37 | <0.001 |  |
|  | All | 486 | 1094 | 0.27 | 0.22-0.33 | <0.001 |  |

This is the S2 Table legend.

MPI-SVaMA: Multidimensional Prognostic Index- Standardized Multidimensional Assessment Schedule for Adults and Aged Persons

Patients were matched, using a 5 to 1 greedy 1:1 PS matching algorithm for: age at SVaMA evaluation, sex, Nursing Care Needs (VIP), cognitive status (VCOG), pressure sores risk (VPIA), activities of daily living (VADL), mobility (VMOB), social support (VSOC) (all MPI-SVaMA domains), the needing of care assistants, the main diagnoses of fractures, cancer, dementia, stroke, hypokinetic syndrome and cardiovascular, respiratory neurological or other diseases and number of all medications prescribed within one year before patient’s enrollment (tertiles)

*Testing whether the effect of statins on mortality risk was differential within MPI-SVaMA grades (effect modifier)
